# Managing a Pregnancy in the Presence of the Rare Blood Group Antibody PP1Pk

**DOI:** 10.1089/whr.2023.0120

**Published:** 2024-04-02

**Authors:** Alexandra Bonmatí-Santané, Roberto Céspedes López, Jehimy Jean Alvarez Saltos, Jordi Calabia Martínez, Cristina Noboa Paez, Jordi Piedra Sánchez, Natalia Visa Figueredo, Anna Maroto González

**Affiliations:** ^1^ Gynecology and Obstetrics Service, Girona, Spain.; ^2^ Department of Hematology, Banc de Sang i Teixits, Girona, Spain.; ^3^ Nephrorolgy Service; Hospital Universitari de Girona Doctor Josep Trueta, Girona, Spain.

**Keywords:** plasmapheresis, anti-PP1Pk, autologous blood donation, miscarriage, p phenotype

## Abstract

Pregnant women with p phenotype, who lack antigens P, P1, and Pk, spontaneously form anti-PP1Pk antibodies whose primary target is the placenta. The risk of miscarriage in these women is 50%–70% and reaches 87% in the second trimester. The therapies aim to reduce the titer of antibodies early in pregnancy. They also have risk of hemolytic transfusion reaction, with very few compatible red blood cell donors in the world. In this study, we present a case of successful pregnancy managed with autologous blood donations and plasmapheresis.

## Introduction

The P and Pk antigens are high prevalence antigens; they are expressed by more than 99% of the world population. Individuals with the p phenotype, who lack P, P1, and Pk antigens (from GLOB and P1Pk blood group systems), spontaneously form IgG and IgM anti-PP1Pk antibodies, without prior contact with the antigen. The prevalence of the p phenotype is estimated to be 5.8 per million.^1–4^

This antibody is associated with hemolytic transfusion reaction, with very few compatible red blood cell donors in the world.^5–7^ In pregnant women with p phenotype, anti-PP1Pk's primary target is the placenta, resulting in placental dysfunction, thereby causing intrauterine growth restriction and fetal death, most likely caused by the cytotoxic effect of IgG and IgM antibodies against the antigens P− and Pk− in placental tissue as early as 5 weeks of gestation. The risk of miscarriage in these women is 50%–70% and reaches 87% in the second trimester. Less risk is described in late pregnancy as risk of hemolytic disease of the fetus and newborn (HDFN).^8^ There is no specific treatment for recurrent abortions linked to anti-PP1Pk.

The therapies aim to reduce the titer of antibodies in early pregnancy stages, as the risk may correlate with the IgG titer. The aim of this article is to present a pregnancy complicated by anti-PP1Pk antibodies successfully managed with autologous blood donations and plasma exchange (PE).^9^

## Case Report

A 33-year-old healthy Caucasian woman was referred to our center at 8 weeks of gestation. She had been evaluated at another institution after one spontaneous miscarriage at 15 weeks of gestation. Chromosomal abnormalities, acquired thrombophilia, uterine malformations, and endocrine pathology were excluded. In the first trimester of her second pregnancy, anti-PP1Pk was identified by an indirect antiglobulin test, and the antibody titer was 128. The 2-mercaptoetanol technique revealed a mixture of IgG and IgM antibodies. Occult alloantibodies were ruled out performing differential alloadsortions.

An ultrasound scan confirmed a viable fetus at 10 weeks of gestation. A multidisciplinary team, including obstetricians, hematologists from the transfusion-medicine department, anesthesiologists, neonatologists, and nephrologists, reviewed the literature to find the best treatment for the patient's pregnancy and delivery.

Relatives were tested as potential donors, but they did not share the same blood phenotype. Moreover, a local and national search for appropriate units of red blood cells did not locate compatible units. The patient was encouraged to donate autologous blood to prepare for the possibility of hemorrhage.

### Treatment with PE

After an extensive review, medical treatments such as aspirin, prednisolone, low-molecular-weight heparin, progesterone, or dydrogesterone were considered.^3,10^ However, to rapidly decrease the titers, a protocol based on PE, without associated immunoglobulin, was accepted by the multidisciplinary team.^11–14^ The limit titer is not clear in literature, however, a titer of 32 was found to be a safe objective.^15^ A tunneled 11F jugular catheter in the 11th week of gestation was placed. A total of 52 PE sessions were completed, using 2500 mL of 5% human albumin until the 21st week; an adjustment was needed to replace the volume to 2700 mL of albumin due to the weight gain during pregnancy, coinciding with an increase in anti-PP1Pk levels to 1:32.

**FIG. 1.**
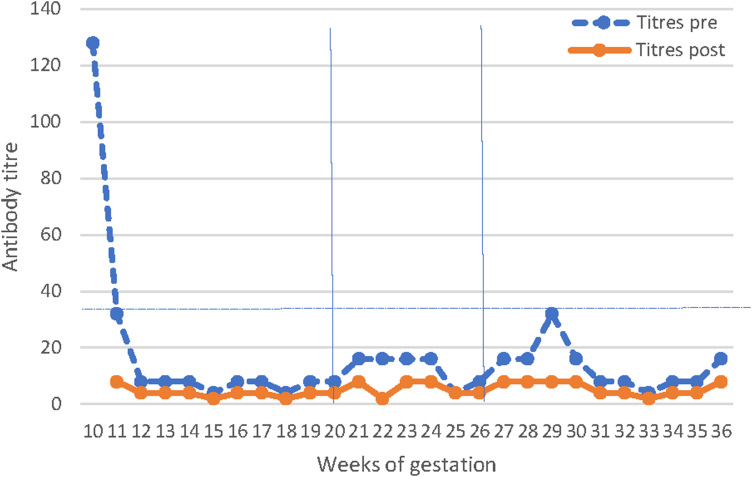
Antibody titers before and after each plasma exchange. A titer of less than 32 as an objective. Three sessions per week until the 20th week. Two sessions per week until the 26th week and one maintenance session a week until the 36th week.

The Plasauto Σ™ system was used with a Plasmaflo filter OP-08W (L), the blood flow rate was 120 mL/min, a replacement rate of 31.2 mL/min, and a variable session duration of 80–100 minutes. PE was performed using 5% human albumin, and regional circuit anticoagulation was achieved using sodium citrate and calcium reinfusion. The antibody titer rapidly decreased with the first PE session and was maintained at less than 32 throughout the remainder of the pregnancy (Fig. 1). Three sessions per week were conducted until the 20th week of gestation, after which it was reduced to two sessions per week. Finally, at the 26th week of gestation, one maintenance session a week was conducted until reaching the 36th week. A final PE was performed in the 36th week of gestation before admission for labor induction.

Each of these sessions was complemented with abdominal ultrasound exploration to confirm fetal viability and to take samples for antibody titration measurement before and after each PE. No complications were observed, and the patient experienced one episode of unrelated otitis media, which required oral antibiotic treatment in the 26th week of gestation. Sessions were well-tolerated always in supine position; no additional strategies were necessary.

### Autologous blood donation

Three uneventful donations of 470 mL were made during the pregnancy, at 14, 27, and 34 weeks of gestation (Table 1). A second scheduled donation at 24 weeks was refused because of hemoglobin less than 10 g/dL. The first two donations were kept frozen in a national bank, whereas the last one was kept in the hospital. The patient was given oral iron supplementation (200 mg of ferrous sulfate each day) and intravenous iron (1000 mg of carboxymaltose iron) after each blood collection. Moreover, folic acid and vitamin B12 were added as supplements. The fetus was monitored by serial ultrasound biometries and Doppler examinations (peak systolic velocity in the middle cerebral artery) aimed at diagnosing fetal growth restriction or fetal anemia (Table 2).

**Table 1.** Maternal monitoring. Three donations: 14, 27, and 33 weeks of gestation

Donation 1						Donation 2						Donation 3					
Pre-Hb	11.7	Post-Hb	10.5	Fetal P	—	Pre-Hb	11.8	Post-Hb	11.5	Fetal P	65	Pre-Hb	11.5	Post-Hb	11.2	Fetal P	40
Pre-Ht	34.6	Post-Ht	32	Anemia	—	Pre-Ht	36	Post-Ht	35	Anemia	No	Pre-Ht	35	Post-Ht	34	Anemia	No
Ferritin	38	Ferritin				Ferritin	39	Ferritin	19			Ferritin	17	Ferritin	51		

Hb (g/dL), Ht (%) and ferritin (ng/mL) levels before (1 week previous) and after (2–3 weeks after) donations.

Hb, hemoglobin; Ht, hematocrit.

**Table 2.** Fetal monitoring. Two donations during third trimester and delivery

	Donation 2	Donation 3	Delivery
Fetal percentile	65	40	38
Fetal anemia	no	no	no

Fetal percentile and fetal anemia (peak systolic velocity in the middle cerebral artery; Multiple of the Median (MoMs) of maximum velocity).

### Delivery

Induction of labor was indicated at 37 weeks of gestation, upon agreement between the patient and the multidisciplinary team. A cesarean section was performed due to a failed induction with minimal blood loss. A healthy female newborn of 2732 g (38 percentile) was born without anemia. Her blood group was B RhD+, direct Coombs was negative, and nonirregular antibodies were found in the newborn serum. The baby's phenotype was heterozygous for PP1Pk blood group.

## Discussion

Pregnant women with the p phenotype present several challenges related to severe hemolytic transfusion reactions, recurrent spontaneous early abortions, as well as second-trimester abortions. Lower risks are described in late pregnancy as well as low risk of HDFN.^3^ A different characteristic from other alloantibodies is their natural development, without prior contact with the antigen. In these pregnancies, a multidisciplinary coordination between the obstetric team, the neonatal unit, anesthesiologists, the transfusion-medicine department, and blood center is required for the development of a care plan. This plan should include the implementation of an early therapeutic protocol to decrease antibody titers, blood collection from the mother, close monitoring of the fetus and mother, and plan for a safe approach for delivery, neonatal management, and preparation for hemorrhage.

Maternal rare antibodies against high prevalence antigens such as PP1Pk may be considered in the differential diagnosis when an irregular antibody is present along with history of miscarriage. Although there are few available data and there is no specific treatment for the recurrent abortion risk, several studies have demonstrated that the implementation of an early therapeutic protocol would reduce the risk.^9,15^ Successful treatments based on anti-inflammatory and immunosuppressive effects with corticosteroids or low-molecular-weight heparin, aspirin, and progesterone or dydrogesterone have been published, with lower titers at early pregnancy stages. PE has been suggested as a potential treatment option, with or without immunoglobulins (to avoid rebound phenomenon), to keep antibody titer low and maintain placental function. Our patient was treated with plasmapheresis alone, the titer rapidly decreased and maintained at less than 32 throughout the remainder of the pregnancy.

The timing of the therapy initiation is extremely important; however, the duration of the therapy, especially after week 20, is still unclear. In our case, the multidisciplinary team decided to continue with one maintenance session until the end of pregnancy to avoid placental dysfunction. No major effects as a result of this therapy were observed in our patient.

Obstetric hemorrhage is a leading cause of maternal and perinatal mortality and red blood cell transfusions may be an important part of the treatment. Women with rare blood groups may find few compatible red blood cell donors. Although autologous blood donation is not recommended during pregnancy, it should be considered in exceptional circumstances such as pregnant women with rare antibodies. Studies about storage and use of autologous blood have demonstrated the safety and efficiency of a program of autologous blood donation from the second trimester of pregnancy, without important effects on the fetus or the mother, except for iatrogenic anemization. Scheduled blood donations may be encouraged if no possible family donors are found or no previous donation has been recollected.^7^

## Conclusion

Our case is an example of a successful pregnancy of a p phenotype pregnant woman (with anti-PP1Pk antibodies) diagnosed in the first trimester. The exceptional nature of this phenotype limits the data available. The main complications of p phenotype pregnant women are high risk of repeated miscarriages and hemolytic transfusion reaction. The establishment of a multidisciplinary and an early-onset program is essential to maintain the pregnancy and to guarantee the safety of both the mother and the fetus. PE can rapidly decrease titers and it is a safe procedure during pregnancy. Although timing and duration are controversial, monitoring titers and keeping them lower than 1:32 might be the objective. Autologous blood donation could be an ideal alternative for pregnant women with p phenotype to ensure the availability of blood transfusions to the mother, the fetus, or newborn if needed, and planning a delivery in case of maternal hemorrhage.

It is important to encourage patients with rare blood types to join a Rare Blood Donor Program and to donate regularly while they are healthy for cryogenic storage and create a stockpile for future pregnancies or procedures.

## Abbreviations Used

Hb: hemoglobin
HDFN: hemolytic disease of the fetus and newborn
Ht: hematocrit
PE: plasma exchange
